# Author Response: Retina Real-World Outcomes After Switch From Aflibercept to Faricimab in Eyes With Diabetic Macular Edema

**DOI:** 10.1167/iovs.66.11.51

**Published:** 2025-08-25

**Authors:** Kim Lien Huber, Irene Steiner, Andreas Pollreisz

**Affiliations:** 1 Medical University of Vienna, Department of Ophthalmology, Vienna, Austria; 2 Medical University of Vienna, Center for Medical Data Science, Institute of Medical Statistics, Vienna, Austria. E-mail: andreas.pollreisz@meduniwien.ac.at.

We read with interest the letter to the *Investigative Ophthalmology & Vision Science* editor by Yang Yang and Junyan Zhang regarding our article “Real-World Outcomes After Switch From Aflibercept to Faricimab in Eyes With Diabetic Macular Edema.”^1^

The purpose of our retrospective, interventional consecutive case series was to assess the anatomic and functional outcomes in eyes with diabetic macular edema (DME) switched from intravitreal aflibercept to faricimab in a real-world setting.

In their letter, Yang and Zhang raise the issue of selection bias. In our study, all patients were selected consecutively without exception at a single center based on rigorously defined and described inclusion and exclusion criteria, aligned with the study objectives. Although the imbalance in observed baseline factors should be considered low, all potential bias cannot be eliminated owing to the nature of the study design.

Further, we provide corrected values to the recalculations performed by Yang and Zhang.

In Table 1 of the article, descriptive statistics of study eyes are provided, whereby some of the patients were measured on both eyes (52 eyes of 40 patients). As mentioned in the Statistical Methods section, *P* values were calculated by using mixed models (eye-specific variables) or by including one eye per patient (patient-specific variables), to account for the dependencies in the data. The corresponding *P* values are shown in the supplement (Supplemental Table S1).

In the Letter to the Editor, *P* values were calculated from Table 1 in the article by Yang and Zhang. Although the *P* values for the comparison of sex (χ² = 3.91; *P* = 0.048) and the mean duration of aflibercept treatment (*t* =1.91; *P* = 0.062) are reproducible, our calculations yield different results for the mean number of aflibercept injections in the past year (letter to the editor: *t* = −3.49; *P* < 0.001). According to Table 1, the mean number of aflibercept injections of the past year was 3.9 ± 1.8 (responding DME, 26 eyes) vs. 4.9 ± 2.3 (nonresponding DME, 26 eyes). The pooled standard deviation is equal to 2.065, the difference between means is equal to −1. This results in a *t*-value of −1.75 and consequently in a *P* value of 0.087. The manual calculation of the *P* value was confirmed by calculating the *t* test using the dataset (*P* = 0.11, the difference to *P* = 0.087 is owing to rounding). It should be noted, that the *P* values mentioned were calculated under the assumption of independent observations, and are therefore not appropriate, because intrapatient correlations (measurements on both eyes) were not accounted for.

The authors further suggested to include potential confounders in the mixed models. We repeated the analyses by including the variables sex, number of injections in the past year, and duration of aflibercept treatment (years) as additional covariates in the mixed models. The results remained similar (data not shown).

Another concern raised by Yang and Zhang is a missing statistical comparison between responding DME and nonresponding DME group regarding time to retreatment. In our manuscript we provide descriptive statistics and graphical illustration for this main outcome. The STROBE statement does not demand the calculation of *P* values for the primary end point and we do not consider it to provide additional interpretative value for our article.^2^

With respect to the third concern regarding insufficient reporting of *P* values, we explicitly address this issue in the Statistical Methods section as follows: “Due to the exploratory character of the study, no adjustment for multiple testing was conducted. The interpretation of the *P* values was exploratory.” This statement clearly rules out any potential for misinterpretation of *P* values by the readership of this esteemed journal.

In conclusion, our study indicates, with statistical robustness, that after a switch to faricimab, treatment intervals in DME eyes that previously responded to aflibercept could be extended both earlier and longer compared with nonresponders, and the improvement in visual acuity did not differ significantly between the two groups.
